# The Effect of Frequency of Fresh Pasture Allocation on Pasture Utilisation and the Performance of High Yielding Dairy Cows

**DOI:** 10.3390/ani10112176

**Published:** 2020-11-21

**Authors:** Jessica G. Pollock, Alan W. Gordon, Kathryn M. Huson, Deborah A. McConnell

**Affiliations:** 1 School of Biological Sciences, Queens University Belfast, Belfast BT9 7BL, UK; 2 Agri-Food and Biosciences Institute, Large Park, Hillsborough BT26 6DR, UK; kathryn.huson@afbini.gov.uk (K.M.H.); deborah.mcconnell@afbini.gov.uk (D.A.M.); 3 Agri-Food and Biosciences Institute, 18a Newforge Lane, Belfast BT9 5PX, UK; alan.gordon@afbini.gov.uk

**Keywords:** dairy cows, grazing, grazing management

## Abstract

**Simple Summary:**

Pasture remains a large component of the diet in many dairy systems across temperate regions. However, animal performance in pasture-based systems is often limited due to the lower dry matter intake (DMI) achieved within these systems, meaning animals fail to perform to their genetic potential. Providing a high herbage allowance can improve DMI and subsequently animal performance at pasture, however such methods result in reduced pasture utilisation rates, decreasing the cost-effectiveness of the pasture-based system. Methods that support high levels of animal performance whilst maintaining high pasture utilisation efficiency are required to support efficient and sustainable pasture-based systems. Hence, the aim of this study was to investigate the impact of different frequencies of pasture allocation (12, 24 and 36 h (h)) on pasture utilisation and the performance of high-yielding dairy cows. The results from this study indicated that 36 h allocations best supported the performance of primiparous animals. The 36 h allocations were also associated with significantly higher pasture utilisation efficiency than 12 h allocations during the first study period, but this was not replicated in the second period, where utilisation was similar across all treatments.

**Abstract:**

Pasture allocation frequency (PAF) can influence pasture availability and grazing behaviour, which subsequently may impact on animal performance. Limited research to-date has investigated grazing management methods to improve the performance of high production dairy cows whilst also achieving high grass utilisation rates. This study evaluated the effect of three different PAF’s (12, 24 and 36 h) on pasture utilisation, the performance of high yielding dairy cows and the interaction with parity. The experiment included two 60-day periods, 90 spring calving dairy cows (27 primiparous animals) in period one and 87 (24 primiparous animals) in period two. The average pre-grazing sward height (11.4 cm) was similar for all treatments in both periods. In period one, pasture utilisation rate was significantly higher (8%) in the 36 h compared to the 12 h treatment. In period two, milk energy output was significantly greater for primiparous animals in the 36 h treatment relative to the other treatments.

## 1. Introduction

Efficient grassland utilisation is a key driver of long-term sustainability in dairy production systems in temperate environments across the globe [1]. Increasing the proportion of grazed grass in dairy cow diets has been associated with both environmental [2] and economic [3] benefits while meeting a growing consumer demand for grass-fed dairy produce [4]. Whilst research has highlighted the potential of lactating dairy cows to consume up to 17 kg dry matter (DM) cow^−1^ day^−1^ on pasture only diets [5], achieving this high level of dry matter intake (DMI) from grazed grass can be challenging and requires the effective management of both the plant and animal in response to varying climatic conditions. Within dairy cow grazing systems there are a number of factors which influence DMI, and considerable research has been undertaken to develop appropriate sward and animal management strategies to facilitate high DMI. For example, increasing pasture allocation per cow has been identified to increase animal DMI in both low and high feed supplementation scenarios [6,7,8,9]. However, this management approach is often associated with a reduction in pasture utilisation which can reduce the cost-effectiveness of the system and negatively impact longer-term grass DMI and consequently animal performance, limiting the use of this approach on commercial farms.

Animal grouping strategy is another key component within the animals feeding environment, which can influence feeding behaviour and subsequently DMI [10]. Within their groups, dairy cattle form a hierarchy through dominance establishment [11]. Dominance in dairy herds is predominately positively correlated to live weight and lactation number [11,12,13], resulting in primiparous animals generally being classed as subordinate. Krohn and Konggaard [14] found grouping subordinate primiparous animals separately within an indoor environment increased their DMI by 20% and subsequently increased milk production. Although grouping primiparous animals separately from multiparous animals may support improved performance and health [14,15], limitations on labour and infrastructure often make this impractical in grazing scenarios on commercial farms. Therefore, grazing herds regularly encompass animals of different parities and production levels, and consequently varying energy demands are common.

To ensure the cost-effectiveness and long-term sustainability of grazing systems for high-yielding dairy herds, there is a need to develop new management strategies which can facilitate high levels of herbage DMI whilst simultaneously maintaining pasture utilisation. Such strategies must be able to achieve these outcomes within mixed-parity herds, where individual cow energy demands are variable, and herd hierarchy can negatively affect the performance of primiparous subordinate cows.

Pasture allocation frequency (PAF) is a management strategy which to-date has received relatively little attention in grazing systems. This approach creates short-term differences in both grass availability and inter-animal competition for resources, potentially impacting grazing behaviour and herbage DMI. Previous indoor studies have found reducing the feeding frequency of a total mixed ration (TMR) from five times to once daily [16] and from once daily to alternative day feeding [17], resulted in an increase in DMI and subsequently improved animal performance due to reduced disruption to the animals natural feeding pattern. However, previous literature on PAF has been inconsistent. In an initial experiment, Dalley, Roche [18] observed reducing PAF from six to one daily allocation improved milk yields of lactating dairy cows however a subsequent experiment observed milk yields were similar for animals offered one or six daily pasture allocations. In contrast, Abrahamse, Dijkstra [19] observed reduced animal performance through a lower milk yield when PAF was reduced from one daily allocation to offering fresh pasture every four days. Within commercial intensive dairy grazing systems, fresh pasture is conventionally allocated either once or twice daily post milking. Although research has investigated very high frequency [18] and low frequency [19] allocations, PAF’s typically used within commercial farming systems are seldom investigated in research trials. In addition, studies to date where the experimental treatment was variable PAF have offered animals higher pasture allowances (>40 kg DM cow^−1^ day^−1^) than would be allocated in commercial settings (approx. 15–20 kg DM cow^−1^ day^−1^) resulting in high post-grazing sward heights, and limiting the inter-animal competition for resources often witnessed in intensive grazing systems on commercial farms, and resulting in poor pasture utilisation rates.

Hence, the objective of this study was to assess the impact of frequency of fresh pasture allocation within typical commercial practices on pasture utilisation efficiency, the performance of high-yielding lactating dairy cows and the interaction between PAF and animal parity group.

## 2. Materials and Methods

The experiment was conducted at the Agri-Food and Biosciences Institute, Hillsborough, Northern Ireland (54°27′ N; 06°04′ W) during summer 2018. Experimental procedures in this study were conducted under an experimental license granted by the Department of Health, Social Services and Public Safety for Northern Ireland in accordance with the Animals Scientific Procedures Act 1986.

### 2.1. Experimental Design

The experiment consisted of two experimental periods both lasting sixty days: period one (P1; 11 May–10 July) and period two (P2; 11 August–10 October). Experimental animals were housed full-time for 25 days (11 July–5 August; non-experimental period) due to a prolonged period of low rainfall that resulted in a shortage of grass on the experimental areas. Mean monthly rainfall recorded at the experimental site was less than the five-year average (Table 1). During June and July 2018 at the experimental site average daily hours of sunshine were greater compared to the five-year average. Similarly, average temperature was higher in May, June, July and August during 2018 relative to the average for the previous five years. Contrastingly, average temperature was lower in 2018 in September and October compared to the five-year average between 2013 and 2017 (Table 1).

Ninety spring calving dairy cows comprising of 66 Holstein cattle and 24 Holstein × Jersey crossbred animals were allocated to one of three treatment groups. Each treatment group (n = 30) consisted of nine primiparous and 21 multiparous animals, mean calving date and lactation number for all treatments was the 4th of February (standard deviation (s.d)., 18.3 d) and of 2.4 (s.d., 1.31), respectively. Treatments were balanced for breed, pre-experimental milk yield [mean 37.2 kg cow^−1^ day^−1^, (s.d., 7.87 kg)], live weight [mean 606 kg, (s.d. 62.1 kg)], body condition score [mean 2.42, (s.d., 0.157)], milk predicted transmitting ability (PTA) [mean 200 kg, (s.d. 141.9 kg)] and kilograms of fat plus protein PTA [mean 26.3 kg, (s.d. 7.09 kg)]. Balanced groups were assigned to one of the three pasture allocation treatments; allocated fresh pasture every: 12 h (h), 24 h or 36 h.

Animals grazed part time from 12 April and commenced full time grazing on 22 April. Animals were divided into their treatment groups on 4 May and had a seven-day adaption period prior to measurements starting.

Throughout the grazing periods animals were offered a concentrate (formulation shown in Appendix A) twice daily during milking via individual concentrate feeders. During period one concentrate was allocated at a rate of 4.5 kg cow^−1^ day^−1^ fresh weight (FW) and 6 kg cow^−1^ day^−1^ FW, for primiparous and multiparous animals, respectively. During period two animals were allocated 5 kg cow^−1^ day^−1^ FW and 6.5 kg cow^−1^ day^−1^ FW for primiparous and multiparous animals, respectively (Table 1). Grazing concentrates offered throughout the study had a mean crude protein, acid detergent fibre, neutral detergent fibre, ash and gross energy concentrations of 187 (s.d., 3.0), 159 (s.d., 3.7), 321 (s.d., 11.3) and 75.8 (s.d., 1.59) g kg^−1^ DM and 18.4 (s.d., 0.05) MJ kg^−1^ DM, respectively.

During the non-experimental period animals were housed in their treatment groups and offered common levels of silage and concentrates. Animals remained in the same treatment groups during P2 however, one Holstein parity one animal was removed from each group (n = 29) due to an ill health animal and to ensure treatment groups remained balanced. Treatments groups had a mean milk yield of 27.6 kg cow^−1^ day^−1^ (s.d., 7.40 kg) and a mean of 2.5 lactations (s.d., 1.30) prior to P2 commencing. Once full-time grazing occurred (5 August) all silage was removed from the diet.

### 2.2. Grazing Management

**Figure 1:**
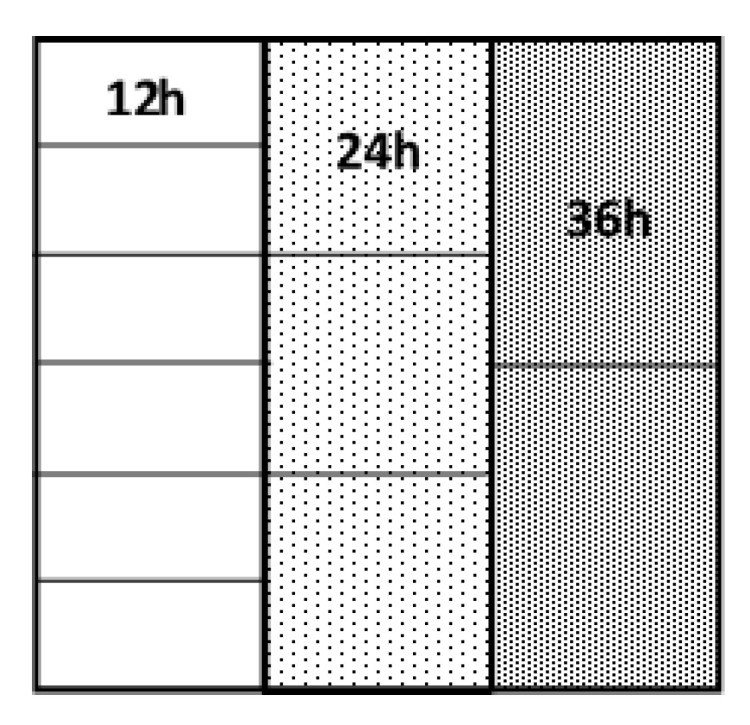
Diagram of 72 h grazing block with six 12 h (0.14 ha), three 24 h (0.28 ha) and two 36 h (0.42 ha) paddocks.

Prior to the experiment commencing all swards were grazed and received 34.5 kg of nitrogen (N) ha^−1^ as urea. The primary experimental area consisted predominately of perennial ryegrass (*Lolium perenne* L.) swards with an average age of five years. The soil type was a slightly gleyed sandy clay-loam (48% sand, 31% silt and 21% clay) overlying Silurian shale (greywacke) till. To account for any geomorphological variation across the experimental area the total area was subdivided into blocks, with individual blocks containing paddocks allocated to each treatment. A total grazing area of 15.12ha and 17.64ha was subdivided into six primary grazing blocks and seven primary grazing blocks, each 2.52 ha in size, in P1 and P2, respectively. Each block consisted of three 0.84 ha plots with each plot comprising of either: six 12 h paddocks (0.14 ha each), three 24 h paddocks (0.28 ha each) or two 36 h paddocks (0.42 ha each; Figure 1). Plots were randomly allocated within the blocks and animals grazed in as close proximity as possible. Animals grazed paddocks in a sequence, once paddocks were grazed, they were then rested for a number of days, this is known as rotational grazing system. Fertiliser in the form of calcium ammonium nitrate (CAN) was applied after each experimental grazing with 41 kg N ha^−1^ in rotation one, 35 kg N ha^−1^ in rotation two, three and four, and 29 kg N ha^−1^ in rotation five and six. Grazing blocks were sown on the same day with a total of 204 kg N ha^−1^ applied during experimental period. Fertiliser was pre-weighed for each individual paddock prior to application by a tractor mounted fertiliser distributer (Vicon, St Helens, UK).

Grass was allocated at a rate of 15 kg DM cow^−1^ day^−1^ throughout the study. Target pre and post grazing heights across all treatments were 10.5 cm (equivalent to 3200 kg DM ha^−1^) and 4.4 cm (equivalent to 1700 kg DM ha^−1^), respectively. Animals in the 24 h treatment were offered fresh pasture after afternoon milking. The 12 h treatment were offered fresh pasture after both morning and afternoon milking while fresh pasture was offered after alternating morning and afternoon milkings for the 36 h treatment. There were four complete rotations in P1 and three in P2. Three primary blocks were topped in P1 after rotation 4 and four primary blocks were topped in P2 after rotation 1 using a disc mower (Lely, Splendimo 320, Maassluis, NL, USA) to a height of approximately 4.0 cm to maintain sward quality. Paddocks within blocks were topped on the same day. If post grazing targets were not met non-experimental animals were used to graze these areas in the immediate 24 h following the grazing by experimental animals.

### 2.3. Sward Measurements

Pre- and post-grazing pasture heights were determined using rising plate meter (RPM; Jenquip EC10 Electronic Platemeter, Feilding, New Zealand) for each paddock. Measurements were taken in a ‘W’ formation across each paddock with 20, 40 and 60 measurements for the 12 h, 24 h and 36 h treatments, respectively. Pasture utilisation was expressed as the ratio of pasture consumed to pasture available (above 4.0 cm) calculated using the following equation:

(1) $$\frac{Pre-grazing\;height\;\left(cm\right)-Post-grazing\;height\;\left(cm\right)}{Pre-grazing\;height\;\left(cm\right)-4.0}$$

Additionally, a representative pasture sample was harvested twice a week from each treatment using electric hand shears (BOSCH, Sebnitz, Germany), cut to a height of 4 cm. Samples were harvested from the appropriate paddocks immediately prior to grazing. Near infrared spectroscopy (NIRS) analysis determined DM content, crude protein (CP), water soluble carbohydrate (WSC), acid detergent fibre (ADF) and metabolisable energy (ME) of the sample using the methodology described by Park, Agnew [20] for grass silage, with a fresh grass calibration equation. Concentrate samples were collected weekly and bulked for each two consecutive weeks. Bulked samples were oven dried at 60 °C for 48 h and subsequently analysed for ADF, neutral detergent fibre (NDF), ash, nitrogen (N), gross energy (GE) and starch concentration by standard wet chemistry analytical techniques as described by Dale, Laidlaw [9].

### 2.4. Animal Measurements

Cows were milked twice daily, between 05.30 and 07.30, and 15.00 and 17.00. Individual cow milk yields (kg) were recorded at each milking. Milk fat, protein and lactose contents were determined weekly from milk samples collected during two consecutive milkings each week. Milk samples were analysed using an infrared milk analyser (Milkoscan Model 605; Foss Electric, Hillerod, Denmark). Weighted milk composition for the 24 h period was determined using the average daily milk yield of the previous seven days. Milk energy concentrations (MJ kg^−1^) were calculated using the equations of Tyrrell and Reid [21].

Milk energy = (fat × 0.0384) + (protein × 0.0223) + (lactose × 0.0199) − 0.108(2)

Milk energy output = milk energy content × average daily yield for previous 7 days(3)

Individual animal liveweight (LW) was recorded after each milking using an automatic weighbridge (BioControl, Rakkestad, Norway). Body-condition score (BCS) was estimated fortnightly using a five-point body condition scoring system [22]. Change in LW and BCS over the course of the experiment were calculated as the difference between the final and initial values.

### 2.5. Statistical Analysis

Due to constraints on grazing infrastructure and animal numbers available for the experiment, it was not feasible to establish replicated groups within each grazing treatment. Therefore, individual animals were used as the experimental unit for statistical analysis, as has been the case for similar studies [9,23]. Although there is ongoing discussion around using individual animals as replicates in grazing experiments [24], it has also been demonstrated that the use of multiple smaller replicate groups may not be appropriate when study results are likely to be impacted by changes in grazing behaviours, as small groups of animals behave differently to larger groups [25] which are found in the commercial systems for which the results of this study would apply.

During P2, data from one primiparous animal was excluded from the results due to a chronic illness. In addition, two primiparous animals were removed from their treatment groups during this period and replaced with animals of similar milk yield, parity and live weight in order to maintain balanced groups.

Data was analysed using Genstat (Genstat Sixteenth Edition, Lawes Agricultural Trust, Rothamsted, UK). Data from each period was analysed separately. Mean weekly milk yields, fat concentrations, protein concentrations, fat plus protein yield and ECM yield were analysed using a linear mixed model methodology with a repeated measures design to take account of correlations in individual animal measurements made at the various time points [26]. The restricted maximum likelihood (REML) estimation method was used to fit all effects in each model and the correlation between time points assessed with an antedependence model of order 1. The individual animal was fitted as a random effect in the models with week of measurement fitted as the time factor. The individual effects of week, parity group and treatment together with their interactions were fitted as fixed effects in all models. Grass data was analysed using linear mixed model methodology using REML as the estimation method with block and plot within block fitted as random effects. The individual effects of rotation and treatment and their interaction were fitted as fixed effects. In all cases if the overall model terms in the fixed effects were significant (*p* < 0.05) two-tailed post-hoc tests were conducted between individual effects using the Bonferroni method for multiple comparisons.

The objective of this study was to determine the effect of altering the frequency of fresh pasture allocation on grass utilisation, the performance of high yielding dairy cows and the interaction effect of PAF and parity group. Other than imposing the three different frequencies of fresh pasture allocation, every effort was taken to ensure all groups were treated the same.

## 3. Results

### 3.1. Pasture Quality and Utilisation 

Pre-grazing sward height was similar for all treatments, with an average pre-grazing height of 11.6 cm and 11.3 cm in P1 and P2, respectively (Table 2). In P1, pasture utilisation rate was 8% lower in the 12 h PAF vs. 36 h PAF (*p* = 0.018; Table 2). Correspondingly, there was a significant (*p* = 0.046) effect of PAF on post-grazing sward height, with the 12 h treatment exhibiting a post-grazing residual 0.8 cm higher relative to the 36 h treatment. However, no significant differences in pasture utilisation or post-grazing sward height were observed in P2. Frequency of fresh pasture allocation did not significantly influence mean pre-grazing chemical composition of the pasture in either period (Table 2). Although data from P1 and P2 were not statistically compared there are a several notable differences between pasture quality in both periods. Average DM content was higher in P1 than P2, with an average DM of 200 g kg^−1^ and 160 g kg^−1^ respectively. In addition, grass WSC and ME content were 56 g kg^−1^ DM and 5 MJ kg^−1^ DM lower respectively, in P2 compared to P1. Average CP and ADF contents were 178 g kg^−1^ DM and 193 g kg^−1^ DM respectively in P1. Both components were higher in P2 with average concentrations of 193 g kg^−1^ DM and 310 g kg^−1^ DM for CP and ADF respectively (Table 2).

### 3.2. Animal Performance

Pasture allocation frequency had no effect on milk yield in either period, and average daily milk yield from all treatment groups was 28.8 and 22.2 kg cow^−1^ day^−1^ for P1 and P2, respectively (Table 3). However, daily milk yield in primiparous animals was significantly lower than multiparous animals, with a mean milk yield of 24.3 kg cow^−1^ day^−1^ and 33.4 kg cow^−1^ day^−1^ in P1, respectively (*p* < 0.001). Similarly, in P2, daily milk yield of primiparous animals was significantly (*p* < 0.001) lower than multiparous animals, with a mean milk yield of 19.4 kg cow^−1^ day^−1^ and 25.1 kg cow^−1^ day^−1^, respectively. Interaction effects of treatment and parity (PAF × PG) were not observed for milk yield in either period (Table 3).

Significant treatment effects were not observed for milk fat or protein content as discrete measurements in either period (Table 3). However, in P1 there was a tendency for a higher milk fat plus protein yield at higher PAF’s (*p* = 0.067), with means being the lowest in the 12 h PAF treatment within each parity. Primiparous animals in the 12 h treatment produced a milk fat plus protein yield, 4% and 6% less yield than the 24 h and 36 h treatments, respectively in P1 (Table 3). In P2, these trends were replicated and the between treatment differences were significant (*p* = 0.012). The 12 h treatment similarly exhibiting the lowest milk fat plus protein yield, producing −0.11 kg cow^−1^ day^−1^ less than the 36 h treatment and −0.09 kg cow^−1^ day^−1^ less than the 24 h treatment. In both periods, multiparous animals produced significantly greater milk fat plus protein yield relative to primiparous animals (*p* < 0.001; Table 3). In addition, PAF × PG interactions were not observed for both milk fat plus protein yield in either period (Table 3). However, significant PAF × PG interactions for milk protein content were observed in P1 (*p* = 0.043; Table 3). Primiparous animals in the 12 h treatment produced milk with an average protein content 1.3 g kg^−1^ cow^−1^ day^−1^ lower than primiparous animals in the 24 h treatment (Table 3). Contrastingly, no significant PAF × PG interactions for milk fat or protein content were observed in P2, although both milk fat and protein content were lowest for the primiparous animals in the 12 h treatment, mirroring trends evident in P1 (Table 3).

Treatment effects were not observed for milk energy output in period one and two. However, in P2 PAF × PG interactions showed that milk energy output was significantly higher for primiparous animals in the 36 h treatment group producing on average 10.9 MJ cow^−1^ day^−1^ more than the other two treatments (*p* < 0.001). Multiparous animals in the 24 h treatment group exhibited the highest milk energy output producing on average 4.7 MJ cow^−1^ day^−1^ more relative to the 12 h and 36 h treatments (Table 3), but this was not significantly different to the other treatment groups. Although these significant interactions were not observed in P1, similar trends did exist, with primiparous animals in the 36 h PAF and multiparous animals in the 24 h treatments both exhibiting the highest milk energy output (Table 3).

Animals in the 24 h treatment lost significantly more weight than animals in the 36 h treatment in P1, by an average of 10.2 kg cow^−1^ (*p* = 0.023; Table 3), this significance was not observed in P2. However, in P2, multiparous animals lost significantly more live weight relative to primiparous animals (*p* = 0.002). Interactions of treatment × parity group were not observed for change in animal live weight in either period, values for the primiparous and multiparous animals were −8.8 kg cow^−1^ and −8.1 kg cow^−1^ respectively for P1, and 4.8 kg cow^−1^ and −8.4 kg cow^−1^ respectively for P2.

## 4. Discussion

### 4.1. Pasture Utilisation and Quality 

The high pasture utilisation efficiency (86%) observed during both periods of this experiment is reflective of an intensive grazing system, comparable to that of other studies offering similar herbage allowances [6,7] and in commercial practice. In the present study the significantly higher pasture utilisation rate (8%) observed in the 36 h treatment in P1 was a direct consequence of the lower post-grazing residual achieved with 36 h allocations (4.7 cm) relative to 12 h allocations (5.5 cm). As all treatment groups received an identical herbage allocation on a per-day basis, it is assumed that a higher DMI within the 36 h treatment caused the decrease in post-grazing sward height. It is postulated that the higher relative pasture allowance offered in the first and second feed of the 36 h treatment facilitated an increased DMI within these feeds, resulting in a higher overall DMI compared to the 12 h treatment. This hypothesis is supported by previous studies that have observed increases in DMI when high pasture allowances were offered [6,7,27]. Although no significant difference in pasture utilisation was observed in P2, animals had a reduced energy demand during this time due to their later stage of lactation, corresponding with lower milk yield and likely lower DMI [9].

Differences in PAF were not found to impact on pasture quality throughout the experiment, this is consistent with results from previous grazing studies that investigated lower [19] and higher [18] frequencies of fresh pasture allocation relative to the present study. The numerically lower nutritional value of the sward during P2 relative to P1, as indicated by lower ME content and higher NDF content, reflects deteriorating sward quality over the grazing season. This common trend is linked to plant physiology and seasonal growth patterns that have been previously described by a number of authors [28,29].

### 4.2. Animal Performance

Average milk production (25.5 kg cow^−1^ day^−1^) throughout the experiment was reflective of high production dairy cows in intensive grazing systems [8,30]. The decreased performance at more frequent PAF observed in the 12 h treatment within the current experiment is comparable to a number of other studies, although most existing research has focussed on very intensive levels of PAF. For example, Dalley, Roche [18] observed that reducing the frequency of fresh pasture from six to one daily allocations improved animal performance, by a 1.0 litre cow^−1^ day^−1^ increase in milk yield (*p* < 0.05). Similarly, Verdon, Rawnsley [23] observed improved animal performance when PAF was reduced from seven to two allocations per day resulting in an increase in fat and protein corrected milk yield (+0.9 kg cow^−1^ day^−1^, *p* < 0.03) and daily milk yield (+1.2 L cow^−1^ day^−1^, *p* < 0.001). This response has also been evident in indoor environments. For example, Phillips and Rind (2001) observed a significant increase in milk yield (+0.66 g cow^−1^ day^−1^) and milk fat yield (44 g cow^−1^ day^−1^) when frequency of TMR feeding was reduced from daily to alternate day feeding. Both Verdon, Rawnsley [23] and Phillips and Rind [17] attributed the reduction in animal performance at higher feeding frequencies to reduced fibre digestion rates due to disturbances in the animals natural feeding pattern. This disturbance likely affects rumen function, impacting on grazing behaviour and ingestion rates. Whilst the disturbance to natural feeding patterns may be smaller for the current study due to the lower PAF imposed relative to the studies of Dalley, Roche [18] and Verdon, Rawnsley [23] this may still have contributed to the lower animal performance witnessed in the 12 h treatment. Previous research has observed milking disrupts animal grazing behaviour with twice daily milking resulting in shorter grazing bouts for animals in the afternoon relative to animals milked once daily [31]. As all treatments were offered fresh pasture after milking it is likely the disruption of the milking process on animals’ natural grazing behaviour, would have impacted all treatments equally. In addition, behaviour studies have observed that lactating dairy cows have several distinct main grazing bouts, with the longest feeding bouts occurring at dusk and dawn [32,33]. Within the 12 h treatment, (relative to the 24 h and 36 h groups) restriction of herbage mass every morning at dawn (before morning milking) may have limited the animal’s ability to graze, therefore impacting the animals’ natural grazing behaviour.

As previously discussed, increasing pasture availability through a high pasture allowance has been found to improve DMI and consequently animal performance [6,7,8]. Curran, Delaby [27] observed a significant increase (*p* < 0.001) in pasture DMI and subsequently animal performance when pasture allowance was increased by 5 kg DM cow^−1^ day^−1^. However, high allocation rates (>20 kg DM cow^−1^ day^−1^) are not commercially viable because they inevitably lead to higher post-grazing sward heights and consequently poor utilisation of the available pasture. In addition, higher post-grazing residuals are associated with deteriorating sward quality throughout the grazing season [34]. Within the present study, the 24 h and 36 h treatments offered a relatively higher pasture allowance in the first feed of each allocation offering 15 kg cow^−1^ and 22.5 kg cow^−1^, respectively. In contrast, 12 h allocations resulted in every feed having less pasture available (7.5 kg DM cow^−1^) due to the incremental delivery of fresh pasture after every milking. In addition, because a limited quantity of pasture is available in each feed within the 12 h treatment animals were forced to graze to low post-grazing sward heights in every feed, this may have resulted in an increased difficulty in harvesting pasture, thus increasing the time and energy expended per bite. Therefore the benefits in animal performance from a reduced PAF are likely because of the combined effects of reduced disturbance to natural feeding behaviour, ease of harvesting and high pasture allowance encouraging DMI during the first (24 h and 36 h) and second (36 h) feed.

The absence of a significant effect on the performance of primiparous animals in the 36 h treatment in P1 may be a consequence of the lower post-grazing residual in this treatment, potentially impacting both milk production and composition. This response of reduced animal performance linked to lower post-grazing residuals is reinforced by the results of Mayne, Newberry [34] who observed a reduction in milk yield of 2.3 kg cow^−1^ day^−1^ and milk protein yield of 66 g cow^−1^ day^−1^ when post-grazing sward height was reduced from 6 cm to 5 cm (*p* < 0.001). Hence maintaining a post-grazing residual greater than 5 cm throughout the grazing season may allow for improved animal performance under the 36 h PAF management however further studies are required to identify the optimal level of pasture allocation for this treatment.

Animals in the 36 h treatment displayed the lowest live weight losses in P1, however the live weight losses experienced were all comparable to those observed with dairy cows in other studies throughout their lactation [30]. In addition, no significant change in live weight was associated with the PAF treatment were observed in P2, nor were there any significant changes in the animals BCS (data not shown).

Similar to the present study, Peyraud, Comeron [35] reported milk yields in primiparous animals are 20 to 30% lower relative to multiparous animals. In the present study, reducing PAF from 12 h or 24 h to 36 h improved the milk energy output of primiparous animals, PAF was not found to significantly impact the milk energy output of multiparous animals. The improved performance of primiparous animals may have been a result of reduced competition for resources, given the lower stocking density (71 livestock units (LU) ha^−1^) in the 36 h grazing paddocks compared to the 24 h (107 LU ha^−1^) and 12 h (214 LU ha^−1^) paddocks. This effect has been observed in indoor environments by DeVries, Von Keyserlingk [36] who identified that increasing feed availability through offering TMR at an increased frequency (once vs. twice daily and twice vs. four times daily), both decreased the displacement of subordinate animals at the feed fence and reduced feed sorting. Feed sorting occurs within both indoor [36] and outdoor [19] feeding environments, where the NDF content is higher in the remaining forage compared to the forage offered as a result of animals preferentially consuming the highest quality forage available. Although feed sorting is inevitable, Phillips and Rind [12] suggested dominant animals may have priority access to the best grazing sites allowing these animals to ingest higher quality pasture. Through a combined effect of a larger grazing area and higher pasture allowance within 36 h treatment during the first two allocations, lower ranking animals would likely experience less competition for high quality grazing sites, and hence increased options for dietary selection across the grazing area. This in turn, is likely to have resulted in the better performance in these animals compared with those on more frequent pasture allocation. These effects are likely to be further exacerbated in swards with greater species diversity than those employed in this current study due to the broader range in sward nutritive value and palatability. However, further investigation of animal grazing behaviour, and associated parity differences, within multi-species swards is required.

In contrast, although all treatments were offered the same pasture allowance over the 72 h grazing block, as previously mentioned animals in the 12 h treatment and to a lesser extent in the 24 h treatment, had a limited quantity of pasture at each allocation due to the phased delivery of feed, limiting opportunities for feed sorting and requiring cows to graze to the target post-grazing residual within each feeding period in order to achieve this intake. Prache and Peyraud [37] have shown animals respond to restricted pasture available through increasing bite mass. In the current study, this response may have presented a further competitive advantage to older heavier multiparous animals within the 12 h and 24 h PAF due to their larger mouth size [23] leading to multiparous animals eating a greater quantities of high quality pasture in comparison with primiparous animals.

## 5. Conclusions

Reducing the frequency of fresh pasture allocation from a typical 12 h or 24 h allocations seen on many commercial farms to 36 h pasture allocations was seen to improve the performance of primiparous animals grazing in mixed-parity herds, relative to 12 h and 24 h allocations. These findings highlight the effect of competition for resources within intensive pasture grazing systems where mixed-parity herds are common. Primiparous animals are most likely to fall lower in the herd hierarchy, and therefore would not gain preferential access to prime grazing areas of swards in highly-competitive grazing environments. Overall, reducing the frequency of fresh pasture allocation from 12 h or 24 h to 36 h allocations created a balance between offering a higher pasture allowance to support animal performance whilst achieving low post-grazing residuals and high levels of grass utilisation, which is a key factor underpinning efficient and economically viable grazing systems. The optimal PAF within individual commercial dairy grazing herds is likely to depend on the herd composition and proportion of primiparous grazing animals. The reduced PAF treatments studied here present a viable method for addressing within herd variation in animal nutritional requirements in grazing systems, to improve animal performance but maintain high pasture utilisation relative to that observed under a typical 12 h or 24 h allocation frequency.

## Figures and Tables

**Table 1:** Rainfall, sunshine hours and average temperature at the Hillsborough site during 2018 and for during previous five years (2013–2017).

Rainfall	May	June	July	August	September	October
5-year average (mm)	67.9	69.3	75.7	110.4	55.7	108.2
2018 (mm)	34.4	24.2	66.4	72.4	34.4	50.0
Sunshine						
5-year average (hours)	6.4	5.4	5.9	5.2	4.3	3.4
2018 (hours)	6.3	8.0	6.6	4.3	3.1	3.6
Temperature						
5-year average (°C)	10.7	13.4	14.8	14.2	12.5	10.0
2018 (°C)	11.8	14.8	15.8	14.4	11.5	9.2

**Table 2:** Effect of frequency of fresh pasture allocation on the mean chemical composition of the pasture on offer, pre- and post-grazing height and pasture utilisation efficiency in P1 and P2.

Pasture factor	Period 1					Period 2				
	12 h	24 h	36 h	SED	*p*-Value	12 h	24 h	36 h	SED	*p*-Value
Pre-grazing sward height (cm)	12.0	11.7	11.1	0.58	0.206	11.2	11.4	11.2	0.36	0.808
Post-grazing sward height (cm)	5.5	5.2	4.7	0.34	0.046	5.2	5.2	5.2	0.23	0.947
Utilisation efficiency	0.83 ^a^	0.85 ^ab^	0.91 ^b^	0.029	0.018	0.85	0.84	0.85	0.034	0.895
Grass Quality:										
DM (g kg^−1^)	205	196	199	5.2	0.092	159	159	162	4.0	0.907
CP (g kg^−1^ DM)	175	177	182	4.9	0.426	193	193	193	6.6	0.744
ADF (g kg^−1^ DM)	276	280	276	4.8	0.542	313	310	308	5.0	0.536
WSC (g kg^−1^ DM)	156	152	152	4.2	0.327	96	100	94	5.4	0.709
ME (MJ kg^−1^ DM)	11.5	11.5	11.5	0.08	0.892	10.9	11.0	11.0	0.09	0.501

12 h = 12 hour allocations. 24 h = 24 hour allocations. 36 h = 36 hour allocations. DM, dry matter. CP, crude protein. ADF, acid detergent fibre. WSC, water soluble sugars. ME, metabolisable energy. Within a row means with different superscript letters differ at (*p* < 0.05) as determined by Bonferroni post-hoc analysis. SED = standard error of differences.

**Table 3:** Effect of frequency of fresh pasture allocation and animal parity on milk production, milk composition and animal live weight for P1.

	Primiparous			Multiparous			SED			
Period 1	12 h	24 h	36 h	12 h	24 h	36 h		Treatment	Parity	Treatment × Parity
Milk yield (kg cow^−1^ day^−1^)	25.0	24.1	23.7	33.5	34.3	32.4	1.52	0.339	<0.001	0.733
Milk fat (g kg^−1^)	38.7	41.3	42.8	39.8	40.5	39.7	1.29	0.466	0.059	0.060
Milk protein (g kg^−1^)	31.7	33.0	32.9	32.8	32.5	32.4	0.54	0.708	0.762	0.041
Milk fat + protein yield (kg cow^−1^ day^−1^)	1.79	1.82	1.90	2.42	2.64	2.46	0.100	0.067	<0.001	0.126
Milk energy output (MJ cow^−1^ day^−1^)	82.2	82.4	84.1	101.9	102.8	100.8	3.3	0.843	<0.001	0.749
Change in live weight (kg cow^−1^)	−6.3	−15.7	−4.7	−10.7	−11.9	−1.7	5.71	0.023	0.816	0.541
Period 2										
Milk yield (kg cow^−1^ day^−1^)	19.1	19.2	19.7	24.5	26.5	24.4	1.51	0.123	<0.001	0.326
Milk fat (g kg^−1^)	43.7	46.0	48.0	46.1	46.2	46.8	2.04	0.105	0.090	0.418
Milk protein (g kg^−1^)	36.5	36.6	37.7	38.3	37.3	38.4	1.14	0.630	0.092	0.463
Milk fat + protein yield (kg cow^−1^ day^−1^)	1.55	1.58	1.69	2.08	2.22	2.16	0.036	0.012	<0.001	0.407
Milk energy output (MJ cow^−1^ day^−1^)	71.2 ^a^	71.8 ^a^	82.4 ^b^	82.6 ^b^	86.8 ^b^	81.6 ^b^	2.98	0.120	<0.001	<0.001
Change in live weight (kg cow^−1^)	8.9	−5.2	10.6	−8.0	−8.6	−8.7	5.02	0.549	0.002	0.242

12 h = 12 hour allocations. 24 h = 24 hour allocations. 36 h = 36 hour allocations. Within a row means are associated with treatment × parity interactions. Means with different superscript letters differ at *p* < 0.05 based on the Bonferroni post-hoc analysis for the effect highlighted in bold for the treatment parity interaction. SED = standard error of differences.

## Appendix A

**Table A1:** Formulation of the grazing concentrate.

Concentrate Ingredient	g kg^−1^ FW
Soya Hulls	187
Maize Meal	160
Wheat	150
Hi-Pro Soya Bean Meal	125
Rape Seed Meal	90
Molaferm	70
Distillers Grain	60
Pollard	57
Citrus-pulp	40
Rumen Protected Fat	20
Minerals/Vitamins	41
